# 4-(Benz­yloxy)phenyl 4-hexa­dec­yloxy-3-meth­oxy­benzoate

**DOI:** 10.1107/S1600536811008403

**Published:** 2011-03-12

**Authors:** Waleed Fadl Ali Al-Eryani, H. T. Srinivasa, S. Jeyaseelan, T. Sadashivaiah, H. C. Devarajegowda

**Affiliations:** a Department of Physics, Yuvaraja’s College (Constituent College), University of Mysore, Mysore 570 005, Karnataka, India; b Raman Research Institute, C.V. Raman Avenue, Sadashivanagar, Bangalore 560 080, Karnataka, India

## Abstract

In the title compound, C_37_H_50_O_5_, the central benzene ring makes dihedral angles of 39.72 (14) and 64.43 (13)° with the benzyl and 3-meth­oxy­benzoate rings, respectively. The crystal structure is stabilized by inter­molecular C—H⋯π inter­actions involving the central benzene ring and the benzene ring closest to the aliphatic chain.

## Related literature

For general background to 4-(hexa­dec­yloxy)-3-meth­oxy­benzoate, see: Parker *et al.* (1977 ▶); Nessim (2011 ▶); Sadashiva & Subba (1975 ▶); Castellano *et al.* (1971 ▶). In a three-ring system, when two rings are linked by a unit which preserves conjugative inter­action and mol­ecular rigidity, the second linking unit can be more flexible, see: Gray (1976 ▶).
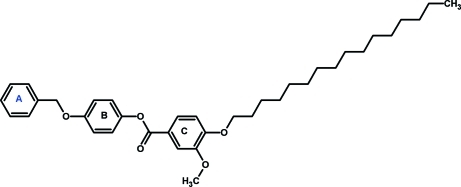


## Experimental

### 

#### Crystal data

C_37_H_50_O_5_
*M* *_r_* = 574.77
Triclinic, 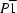

*a* = 5.4507 (2) Å
*b* = 9.7352 (4) Å
*c* = 31.3738 (14) Å
α = 94.155 (4)°
β = 94.261 (4)°
γ = 95.576 (4)°
*V* = 1647.02 (12) Å^3^
*Z* = 2
Mo *K*α radiation
μ = 0.08 mm^−1^
*T* = 293 K
0.22 × 0.15 × 0.12 mm

#### Data collection

Oxford Diffraction Xcalibur diffractometer
Absorption correction: multi-scan (*CrysAlis PRO RED*; Oxford Diffraction, 2010 ▶) *T*_min_ = 0.664, *T*_max_ = 1.000
30991 measured reflections
5841 independent reflections
2558 reflections with *I* > 2σ(*I*)
*R*_int_ = 0.063

#### Refinement

*R*[*F* ^2^ > 2σ(*F* ^2^)] = 0.052
*wR*(*F* ^2^) = 0.131
*S* = 0.91
5841 reflections
379 parameters
H-atom parameters constrained
Δρ_max_ = 0.13 e Å^−3^
Δρ_min_ = −0.16 e Å^−3^

### 

Data collection: *CrysAlis PRO CCD* (Oxford Diffraction, 2010 ▶); cell refinement: *CrysAlis PRO CCD*; data reduction: *CrysAlis PRO RED* (Oxford Diffraction, 2010 ▶); program(s) used to solve structure: *SHELXS97* (Sheldrick, 2008 ▶); program(s) used to refine structure: *SHELXL97* (Sheldrick, 2008 ▶); molecular graphics: *ORTEP-3* (Farrugia, 1997 ▶) and *CAMERON* (Watkin *et al.*, 1993 ▶); software used to prepare material for publication: *WinGX* (Farrugia, 1999 ▶).

## Supplementary Material

Crystal structure: contains datablocks I, global. DOI: 10.1107/S1600536811008403/wn2423sup1.cif

Structure factors: contains datablocks I. DOI: 10.1107/S1600536811008403/wn2423Isup2.hkl

Additional supplementary materials:  crystallographic information; 3D view; checkCIF report

## Figures and Tables

**Table 1:** Hydrogen-bond geometry (Å, °) *Cg*2 and *Cg*3 are the centroids of the C8–C13 and C14–C20 rings, respectively.

*D*—H⋯*A*	*D*—H	H⋯*A*	*D*⋯*A*	*D*—H⋯*A*
C6—H6⋯*Cg*2^i^	0.93	2.97	3.576 (3)	124
C22—H22*B*⋯*Cg*3^ii^	0.96	2.94	3.793 (2)	148

Symmetry codes: (i) 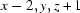; (ii) 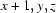.

**Fig. 1.**
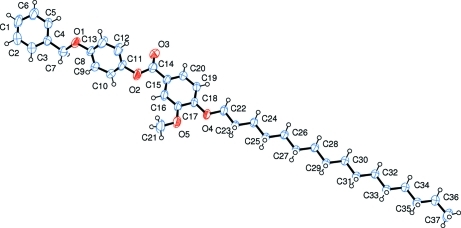
The title molecule with displacement ellipsoids drawn at the 50% probability level. H atoms are shown as small spheres of arbitrary radius.

**Fig. 2.**
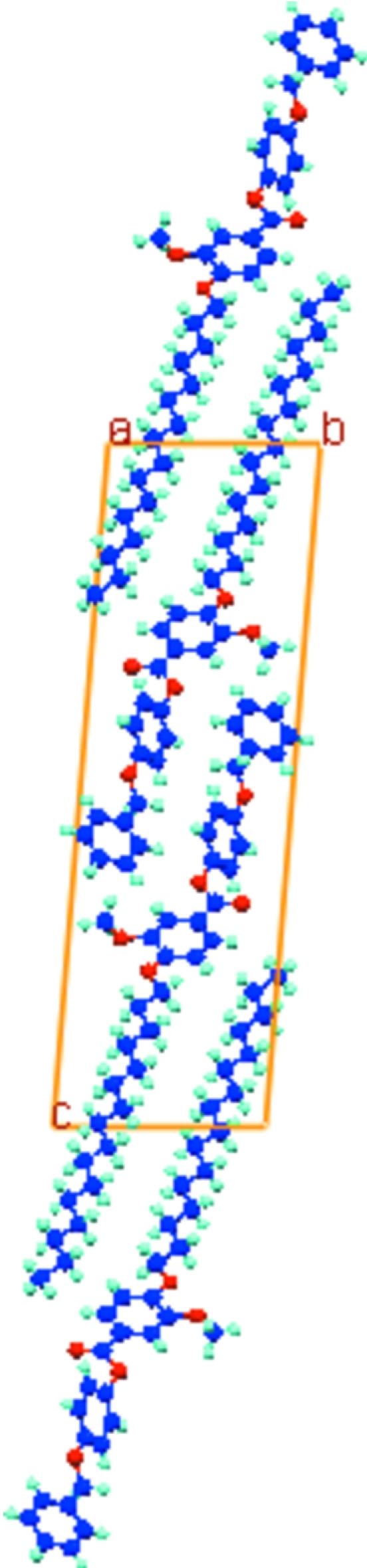
A view of the crystal structure down the a axis.

## supplementary crystallographic information

### Comment

Many organic compounds exhibiting liquid crystalline properties contain two phenyl rings with substituents in the *para* positions. On moving from two-ring mesogens with one linking unit to three-ring mesogens with two linking units, mesophase thermal stabilities are greatly enhanced. In a three-ring system, when two rings are linked by a unit which preserves conjugative interaction and molecular rigidity, the second linking unit can be more flexible (Gray, 1976).

The electron-rich title compound,4-(benzyloxy)phenyl 4-(hexadecyloxy)-3-methoxybenzoate has a long flexible aliphatic chain with a bulky, laterally substituted methoxy group at one end and the other end having a hydrophobic benzyl group. Lateral and terminal substitution lead to a significant change in some of the properties of compounds having medical importance and also in obtaining desired properties in mesogenic materials (Parker *et al.*,1977; Nessim, 2010; Sadashiva *et al.*,1975). With this background, we have synthesized (Castellano *et al.*, 1971) the title compound, 4-(benzyloxy)phenyl 4-(hexadecyloxy)-3-methoxybenzoate, and here we report its crystal structure.

The crystal structure of the title compound contains one molecule in the asymmetric unit (Fig. 1).The dihedral angle between the aromatic rings are: A/B, B/C and A/C 39.72 (14)°, 64.43 (13)° and 24.72 (13)°, respectively. The alkyl chain and aromatic ring C make a dihedral angle of 5.71 (10)°. The packing of the molecules is stabilized by intermolecular C6—H6···Cg2 and C22—H22B···Cg3 interactions, where Cg2 and Cg3 are the centroids of rings B and C, respectively (Table 1). The packing of the molecules in the title structure is depicted in Fig. 2.

### Experimental

A mixture of 4-(hexadecyloxy)-3-methoxybenzoyl chloride (5 mmol) was added to 4-(benzyloxy)phenol (5 ml) in 5 mol of dry dichloromethane. The resultant mixture was stirred at room temperature for 30 min and heated for 10 min at 338 K, then poured into ice-cold hydrochloric acid and extracted with dichloromethane. The combined organic layers were washed with water and dried. Evaporation of the solvent under vacuum yielded a white solid material which was recrystallized from pure dichloromethane at room temperature. The yield was about 92%. M.p. 358 K. Elemental analysis for C_37_H_50_O_5_ requires C 77.31%, H 8.77%. Found C 76.98%, H 8.35%.

### Refinement

All H atoms were placed at calculated positions with C—H = 0.93 Å for aromatic H, 0.97 Å for methylene H and 0.96 Å for methyl H. They were refined using a riding model with *U*_iso_(H) =1.5*U*_eq_(C) for methyl H and 1.2*U*_eq_(C) for all other H.

### Crystal data

**Table:**

C_37_H_50_O_5_	*Z* = 2
*M**_r_* = 574.77	*F*(000) = 624
Triclinic, *P*1	*D*_x_ = 1.159 Mg m^−^^3^
Hall symbol: -P 1	Melting point: 358 K
*a* = 5.4507 (2) Å	Mo *K*α radiation, λ = 0.71073 Å
*b* = 9.7352 (4) Å	Cell parameters from 5841 reflections
*c* = 31.3738 (14) Å	θ = 2.1–25.0°
α = 94.155 (4)°	µ = 0.08 mm^−^^1^
β = 94.261 (4)°	*T* = 293 K
γ = 95.576 (4)°	Prism, colourless
*V* = 1647.02 (12) Å^3^	0.22 × 0.15 × 0.12 mm

### Data collection

**Table:**

Oxford Diffraction Xcalibur diffractometer	5841 independent reflections
Radiation source: Enhance (Mo) X-ray Source	2558 reflections with *I* > 2σ(*I*)
graphite	*R*_int_ = 0.063
Detector resolution: 29.3621 pixels mm^-1^	θ_max_ = 25.0°, θ_min_ = 2.1°
ω scans	*h* = −6→6
Absorption correction: multi-scan (*CrysAlis PRO RED*; Oxford Diffraction, 2010)	*k* = −11→11
*T*_min_ = 0.664, *T*_max_ = 1.000	*l* = −37→37
30991 measured reflections	

### Refinement

**Table:**

Refinement on *F*^2^	Primary atom site location: structure-invariant direct methods
Least-squares matrix: full	Secondary atom site location: difference Fourier map
*R*[*F*^2^ > 2σ(*F*^2^)] = 0.052	Hydrogen site location: inferred from neighbouring sites
*wR*(*F*^2^) = 0.131	H-atom parameters constrained
*S* = 0.91	*w* = 1/[σ^2^(*F*_o_^2^) + (0.0564*P*)^2^] where *P* = (*F*_o_^2^ + 2*F*_c_^2^)/3
5841 reflections	(Δ/σ)_max_ < 0.001
379 parameters	Δρ_max_ = 0.13 e Å^−^^3^
0 restraints	Δρ_min_ = −0.16 e Å^−^^3^

### Special details

**Table:**

Experimental. *CrysAlis PRO*, Oxford Diffraction Ltd., Version 1.171.33.55 (release 05–01–2010 CrysAlis171. NET) Empirical absorption correction using spherical harmonics, implemented in SCALE3 ABSPACK scaling algorithm.Experimental data is IR (KBr) cm^-1^; 2912(C—H aromatic stretch), 2870 (C—H aliphatic stretch), 1730 (C=O stretch),1597 (C=C stretch).
Geometry. All s.u.'s (except the s.u. in the dihedral angle between two l.s. planes) are estimated using the full covariance matrix. The cell s.u.'s are taken into account individually in the estimation of s.u.'s in distances, angles and torsion angles; correlations between s.u.'s in cell parameters are only used when they are defined by crystal symmetry. An approximate (isotropic) treatment of cell s.u.'s is used for estimating s.u.'s involving l.s. planes.
Refinement. Refinement of *F*^2^ against ALL reflections. The weighted *R*-factor *wR* and goodness of fit *S* are based on *F*^2^, conventional *R*-factors *R* are based on *F*, with *F* set to zero for negative *F*^2^. The threshold expression of *F*^2^ > 2σ(*F*^2^) is used only for calculating *R*-factors(gt) *etc*. and is not relevant to the choice of reflections for refinement. *R*-factors based on *F*^2^ are statistically about twice as large as those based on *F*, and *R*- factors based on ALL data will be even larger.

### Fractional atomic coordinates and isotropic or equivalent isotropic displacement parameters (Å^2^)

**Table:**

	*x*	*y*	*z*	*U*_iso_*/*U*_eq_	
O1	−2.0141 (3)	0.22569 (17)	0.48377 (5)	0.0715 (5)	
O2	−1.4349 (3)	0.40474 (17)	0.35750 (5)	0.0774 (6)	
O3	−1.3847 (3)	0.19547 (19)	0.32625 (5)	0.0790 (6)	
O4	−0.6971 (3)	0.60994 (15)	0.22712 (5)	0.0608 (5)	
O5	−0.9578 (3)	0.74931 (17)	0.27583 (5)	0.0829 (6)	
C1	−2.4908 (6)	0.1166 (4)	0.60729 (10)	0.0955 (10)	
H1	−2.6039	0.0818	0.6256	0.115*	
C2	−2.3740 (6)	0.2458 (4)	0.61544 (9)	0.1069 (11)	
H2	−2.4086	0.3007	0.6393	0.128*	
C3	−2.2046 (5)	0.2967 (3)	0.58876 (8)	0.0859 (9)	
H3	−2.1264	0.3859	0.5948	0.103*	
C4	−2.1492 (4)	0.2191 (3)	0.55382 (7)	0.0583 (7)	
C5	−2.2705 (5)	0.0905 (3)	0.54574 (9)	0.0948 (10)	
H5	−2.2375	0.0357	0.5217	0.114*	
C6	−2.4411 (6)	0.0389 (3)	0.57221 (10)	0.1091 (12)	
H6	−2.5222	−0.0496	0.5659	0.131*	
C7	−1.9532 (5)	0.2735 (3)	0.52682 (7)	0.0771 (8)	
H7A	−1.9371	0.3739	0.5296	0.092*	
H7B	−1.7958	0.2432	0.5366	0.092*	
C8	−1.8580 (5)	0.2699 (3)	0.45418 (8)	0.0590 (7)	
C9	−1.6344 (5)	0.3459 (3)	0.46385 (8)	0.0733 (8)	
H9	−1.5769	0.3710	0.4923	0.088*	
C10	−1.4936 (5)	0.3853 (3)	0.43083 (9)	0.0793 (9)	
H10	−1.3412	0.4372	0.4372	0.095*	
C11	−1.5760 (5)	0.3489 (3)	0.38936 (8)	0.0630 (7)	
C12	−1.7929 (5)	0.2679 (3)	0.37930 (7)	0.0756 (8)	
H12	−1.8449	0.2388	0.3509	0.091*	
C13	−1.9343 (5)	0.2295 (3)	0.41190 (8)	0.0780 (9)	
H13	−2.0841	0.1752	0.4052	0.094*	
C14	−1.3455 (5)	0.3188 (3)	0.32803 (8)	0.0597 (7)	
C15	−1.1821 (4)	0.3971 (3)	0.30030 (7)	0.0508 (6)	
C16	−1.1560 (4)	0.5408 (2)	0.30206 (7)	0.0586 (7)	
H16	−1.2479	0.5910	0.3202	0.070*	
C17	−0.9947 (4)	0.6095 (2)	0.27701 (7)	0.0557 (7)	
C18	−0.8549 (4)	0.5336 (2)	0.25018 (6)	0.0488 (6)	
C19	−0.8828 (4)	0.3917 (2)	0.24854 (6)	0.0545 (6)	
H19	−0.7900	0.3409	0.2307	0.065*	
C20	−1.0477 (4)	0.3241 (2)	0.27319 (7)	0.0560 (7)	
H20	−1.0677	0.2278	0.2714	0.067*	
C21	−1.1092 (6)	0.8323 (3)	0.30046 (9)	0.1038 (11)	
H21A	−1.0653	0.9284	0.2968	0.156*	
H21B	−1.0833	0.8164	0.3302	0.156*	
H21C	−1.2801	0.8076	0.2908	0.156*	
C22	−0.5539 (4)	0.5369 (2)	0.19813 (7)	0.0530 (6)	
H22A	−0.6623	0.4775	0.1771	0.064*	
H22B	−0.4480	0.4799	0.2137	0.064*	
C23	−0.4008 (4)	0.6428 (2)	0.17643 (6)	0.0509 (6)	
H23A	−0.2882	0.6983	0.1978	0.061*	
H23B	−0.5088	0.7040	0.1632	0.061*	
C24	−0.2528 (4)	0.5794 (2)	0.14255 (6)	0.0492 (6)	
H24A	−0.3656	0.5235	0.1213	0.059*	
H24B	−0.1446	0.5185	0.1559	0.059*	
C25	−0.0981 (4)	0.6856 (2)	0.12022 (6)	0.0513 (6)	
H25A	−0.2063	0.7487	0.1081	0.062*	
H25B	0.0182	0.7390	0.1415	0.062*	
C26	0.0445 (4)	0.6264 (2)	0.08497 (6)	0.0480 (6)	
H26A	−0.0710	0.5725	0.0637	0.058*	
H26B	0.1546	0.5641	0.0970	0.058*	
C27	0.1956 (4)	0.7352 (2)	0.06299 (6)	0.0481 (6)	
H27A	0.0850	0.7974	0.0511	0.058*	
H27B	0.3103	0.7892	0.0844	0.058*	
C28	0.3407 (4)	0.6787 (2)	0.02756 (6)	0.0490 (6)	
H28A	0.2261	0.6259	0.0059	0.059*	
H28B	0.4501	0.6156	0.0393	0.059*	
C29	0.4934 (4)	0.7886 (2)	0.00630 (6)	0.0495 (6)	
H29A	0.3838	0.8516	−0.0055	0.059*	
H29B	0.6076	0.8415	0.0280	0.059*	
C30	0.6396 (4)	0.7329 (2)	−0.02914 (6)	0.0489 (6)	
H30A	0.7481	0.6692	−0.0175	0.059*	
H30B	0.5253	0.6809	−0.0510	0.059*	
C31	0.7935 (4)	0.8430 (2)	−0.04990 (7)	0.0503 (6)	
H31A	0.9070	0.8954	−0.0280	0.060*	
H31B	0.6848	0.9063	−0.0617	0.060*	
C32	0.9411 (4)	0.7875 (2)	−0.08517 (6)	0.0487 (6)	
H32A	1.0507	0.7247	−0.0733	0.058*	
H32B	0.8277	0.7344	−0.1070	0.058*	
C33	1.0935 (4)	0.8972 (2)	−0.10622 (7)	0.0509 (6)	
H33A	1.2059	0.9507	−0.0844	0.061*	
H33B	0.9837	0.9596	−0.1183	0.061*	
C34	1.2422 (4)	0.8422 (2)	−0.14120 (6)	0.0507 (6)	
H34A	1.1301	0.7857	−0.1624	0.061*	
H34B	1.3560	0.7825	−0.1289	0.061*	
C35	1.3882 (4)	0.9510 (2)	−0.16365 (7)	0.0558 (6)	
H35A	1.2741	1.0081	−0.1773	0.067*	
H35B	1.4953	1.0103	−0.1423	0.067*	
C36	1.5435 (4)	0.8939 (2)	−0.19683 (7)	0.0631 (7)	
H36A	1.4371	0.8313	−0.2174	0.076*	
H36B	1.6620	0.8400	−0.1829	0.076*	
C37	1.6829 (5)	1.0013 (3)	−0.22100 (8)	0.0932 (9)	
H37A	1.7788	0.9560	−0.2412	0.140*	
H37B	1.7910	1.0632	−0.2011	0.140*	
H37C	1.5674	1.0528	−0.2360	0.140*	

### Atomic displacement parameters (Å^2^)

**Table:**

	*U*^11^	*U*^22^	*U*^33^	*U*^12^	*U*^13^	*U*^23^
O1	0.0717 (12)	0.0933 (14)	0.0471 (10)	−0.0190 (10)	0.0214 (9)	0.0101 (9)
O2	0.1010 (15)	0.0651 (12)	0.0736 (12)	0.0027 (10)	0.0536 (11)	0.0179 (10)
O3	0.0947 (14)	0.0650 (13)	0.0781 (12)	−0.0125 (11)	0.0359 (10)	0.0087 (10)
O4	0.0723 (12)	0.0557 (11)	0.0594 (10)	0.0054 (9)	0.0386 (9)	0.0064 (8)
O5	0.1192 (15)	0.0517 (12)	0.0887 (13)	0.0117 (11)	0.0694 (11)	0.0117 (10)
C1	0.107 (3)	0.103 (3)	0.086 (2)	0.009 (2)	0.060 (2)	0.0217 (19)
C2	0.115 (3)	0.121 (3)	0.084 (2)	−0.003 (2)	0.054 (2)	−0.023 (2)
C3	0.094 (2)	0.089 (2)	0.0725 (19)	−0.0078 (18)	0.0354 (18)	−0.0121 (16)
C4	0.0689 (18)	0.0592 (17)	0.0485 (15)	0.0009 (14)	0.0225 (13)	0.0065 (13)
C5	0.124 (3)	0.068 (2)	0.095 (2)	−0.0101 (19)	0.072 (2)	−0.0079 (16)
C6	0.137 (3)	0.072 (2)	0.125 (3)	−0.010 (2)	0.087 (2)	0.005 (2)
C7	0.091 (2)	0.085 (2)	0.0518 (17)	−0.0193 (16)	0.0256 (15)	−0.0008 (14)
C8	0.0557 (17)	0.0704 (18)	0.0519 (17)	−0.0068 (14)	0.0208 (14)	0.0136 (13)
C9	0.084 (2)	0.079 (2)	0.0523 (16)	−0.0247 (17)	0.0274 (15)	−0.0056 (13)
C10	0.086 (2)	0.0706 (19)	0.077 (2)	−0.0273 (16)	0.0371 (18)	−0.0089 (15)
C11	0.072 (2)	0.0627 (18)	0.0576 (18)	−0.0030 (15)	0.0329 (16)	0.0160 (14)
C12	0.0631 (19)	0.120 (2)	0.0447 (16)	0.0001 (18)	0.0124 (14)	0.0197 (15)
C13	0.0581 (18)	0.121 (2)	0.0516 (17)	−0.0179 (17)	0.0087 (15)	0.0165 (16)
C14	0.0623 (18)	0.0673 (19)	0.0499 (16)	−0.0013 (16)	0.0143 (14)	0.0088 (15)
C15	0.0534 (16)	0.0580 (17)	0.0423 (14)	−0.0003 (13)	0.0120 (12)	0.0111 (12)
C16	0.0721 (18)	0.0588 (17)	0.0508 (15)	0.0114 (14)	0.0308 (13)	0.0109 (12)
C17	0.0712 (18)	0.0507 (16)	0.0492 (14)	0.0050 (14)	0.0274 (14)	0.0094 (12)
C18	0.0534 (16)	0.0560 (17)	0.0394 (13)	0.0035 (13)	0.0192 (12)	0.0072 (12)
C19	0.0629 (17)	0.0542 (17)	0.0489 (14)	0.0070 (14)	0.0222 (13)	0.0016 (12)
C20	0.0676 (18)	0.0528 (16)	0.0472 (14)	−0.0040 (14)	0.0166 (14)	0.0036 (12)
C21	0.151 (3)	0.0630 (19)	0.115 (2)	0.0319 (19)	0.088 (2)	0.0190 (17)
C22	0.0544 (16)	0.0575 (16)	0.0498 (14)	0.0072 (13)	0.0201 (13)	0.0060 (12)
C23	0.0556 (16)	0.0503 (15)	0.0500 (14)	0.0074 (12)	0.0190 (12)	0.0074 (11)
C24	0.0491 (15)	0.0520 (15)	0.0493 (14)	0.0067 (12)	0.0173 (12)	0.0076 (11)
C25	0.0521 (16)	0.0518 (15)	0.0521 (14)	0.0040 (12)	0.0190 (13)	0.0061 (12)
C26	0.0486 (15)	0.0483 (14)	0.0487 (14)	0.0038 (12)	0.0144 (12)	0.0064 (11)
C27	0.0493 (15)	0.0478 (14)	0.0485 (13)	0.0040 (12)	0.0149 (12)	0.0035 (11)
C28	0.0498 (15)	0.0484 (15)	0.0504 (14)	0.0036 (12)	0.0141 (12)	0.0060 (11)
C29	0.0524 (15)	0.0472 (15)	0.0502 (14)	0.0033 (12)	0.0153 (12)	0.0042 (11)
C30	0.0499 (15)	0.0515 (15)	0.0465 (14)	0.0029 (12)	0.0140 (12)	0.0045 (11)
C31	0.0509 (15)	0.0492 (15)	0.0520 (14)	0.0024 (12)	0.0152 (12)	0.0041 (11)
C32	0.0510 (15)	0.0474 (15)	0.0488 (14)	0.0037 (12)	0.0137 (12)	0.0036 (11)
C33	0.0535 (15)	0.0478 (15)	0.0529 (14)	0.0032 (12)	0.0170 (12)	0.0042 (11)
C34	0.0505 (15)	0.0524 (15)	0.0500 (14)	0.0031 (12)	0.0135 (12)	0.0020 (11)
C35	0.0557 (16)	0.0552 (16)	0.0586 (15)	0.0049 (13)	0.0200 (13)	0.0044 (12)
C36	0.0640 (17)	0.0678 (18)	0.0587 (15)	0.0028 (14)	0.0225 (14)	0.0002 (13)
C37	0.100 (2)	0.097 (2)	0.092 (2)	0.0082 (18)	0.0546 (18)	0.0211 (17)

### Geometric parameters (Å, °)

**Table:**

O1—C8	1.369 (2)	C22—H22A	0.9700
O1—C7	1.401 (2)	C22—H22B	0.9700
O2—C14	1.352 (3)	C23—C24	1.514 (3)
O2—C11	1.414 (2)	C23—H23A	0.9700
O3—C14	1.195 (3)	C23—H23B	0.9700
O4—C18	1.364 (2)	C24—C25	1.514 (3)
O4—C22	1.436 (2)	C24—H24A	0.9700
O5—C17	1.360 (2)	C24—H24B	0.9700
O5—C21	1.437 (3)	C25—C26	1.511 (3)
C1—C6	1.349 (4)	C25—H25A	0.9700
C1—C2	1.351 (4)	C25—H25B	0.9700
C1—H1	0.9300	C26—C27	1.515 (3)
C2—C3	1.373 (3)	C26—H26A	0.9700
C2—H2	0.9300	C26—H26B	0.9700
C3—C4	1.355 (3)	C27—C28	1.513 (3)
C3—H3	0.9300	C27—H27A	0.9700
C4—C5	1.356 (3)	C27—H27B	0.9700
C4—C7	1.493 (3)	C28—C29	1.514 (3)
C5—C6	1.375 (3)	C28—H28A	0.9700
C5—H5	0.9300	C28—H28B	0.9700
C6—H6	0.9300	C29—C30	1.514 (3)
C7—H7A	0.9700	C29—H29A	0.9700
C7—H7B	0.9700	C29—H29B	0.9700
C8—C9	1.365 (3)	C30—C31	1.511 (3)
C8—C13	1.378 (3)	C30—H30A	0.9700
C9—C10	1.388 (3)	C30—H30B	0.9700
C9—H9	0.9300	C31—C32	1.514 (3)
C10—C11	1.355 (3)	C31—H31A	0.9700
C10—H10	0.9300	C31—H31B	0.9700
C11—C12	1.359 (3)	C32—C33	1.509 (3)
C12—C13	1.377 (3)	C32—H32A	0.9700
C12—H12	0.9300	C32—H32B	0.9700
C13—H13	0.9300	C33—C34	1.509 (3)
C14—C15	1.487 (3)	C33—H33A	0.9700
C15—C20	1.368 (3)	C33—H33B	0.9700
C15—C16	1.390 (3)	C34—C35	1.510 (3)
C16—C17	1.379 (3)	C34—H34A	0.9700
C16—H16	0.9300	C34—H34B	0.9700
C17—C18	1.396 (3)	C35—C36	1.499 (3)
C18—C19	1.371 (3)	C35—H35A	0.9700
C19—C20	1.379 (3)	C35—H35B	0.9700
C19—H19	0.9300	C36—C37	1.514 (3)
C20—H20	0.9300	C36—H36A	0.9700
C21—H21A	0.9600	C36—H36B	0.9700
C21—H21B	0.9600	C37—H37A	0.9600
C21—H21C	0.9600	C37—H37B	0.9600
C22—C23	1.497 (3)	C37—H37C	0.9600
			
C8—O1—C7	117.87 (18)	C23—C24—C25	113.47 (17)
C14—O2—C11	119.68 (19)	C23—C24—H24A	108.9
C18—O4—C22	117.86 (17)	C25—C24—H24A	108.9
C17—O5—C21	117.79 (19)	C23—C24—H24B	108.9
C6—C1—C2	119.1 (3)	C25—C24—H24B	108.9
C6—C1—H1	120.5	H24A—C24—H24B	107.7
C2—C1—H1	120.5	C26—C25—C24	115.03 (18)
C1—C2—C3	120.5 (3)	C26—C25—H25A	108.5
C1—C2—H2	119.7	C24—C25—H25A	108.5
C3—C2—H2	119.7	C26—C25—H25B	108.5
C4—C3—C2	121.2 (3)	C24—C25—H25B	108.5
C4—C3—H3	119.4	H25A—C25—H25B	107.5
C2—C3—H3	119.4	C25—C26—C27	113.78 (17)
C3—C4—C5	117.6 (2)	C25—C26—H26A	108.8
C3—C4—C7	120.2 (2)	C27—C26—H26A	108.8
C5—C4—C7	122.2 (2)	C25—C26—H26B	108.8
C4—C5—C6	121.6 (3)	C27—C26—H26B	108.8
C4—C5—H5	119.2	H26A—C26—H26B	107.7
C6—C5—H5	119.2	C28—C27—C26	114.88 (17)
C1—C6—C5	120.0 (3)	C28—C27—H27A	108.5
C1—C6—H6	120.0	C26—C27—H27A	108.5
C5—C6—H6	120.0	C28—C27—H27B	108.5
O1—C7—C4	110.1 (2)	C26—C27—H27B	108.5
O1—C7—H7A	109.6	H27A—C27—H27B	107.5
C4—C7—H7A	109.6	C27—C28—C29	114.26 (17)
O1—C7—H7B	109.6	C27—C28—H28A	108.7
C4—C7—H7B	109.6	C29—C28—H28A	108.7
H7A—C7—H7B	108.2	C27—C28—H28B	108.7
C9—C8—O1	124.8 (2)	C29—C28—H28B	108.7
C9—C8—C13	119.1 (2)	H28A—C28—H28B	107.6
O1—C8—C13	116.0 (2)	C30—C29—C28	114.61 (17)
C8—C9—C10	119.3 (2)	C30—C29—H29A	108.6
C8—C9—H9	120.3	C28—C29—H29A	108.6
C10—C9—H9	120.3	C30—C29—H29B	108.6
C11—C10—C9	120.7 (2)	C28—C29—H29B	108.6
C11—C10—H10	119.7	H29A—C29—H29B	107.6
C9—C10—H10	119.7	C31—C30—C29	114.33 (17)
C10—C11—C12	120.7 (2)	C31—C30—H30A	108.7
C10—C11—O2	117.3 (2)	C29—C30—H30A	108.7
C12—C11—O2	121.9 (2)	C31—C30—H30B	108.7
C11—C12—C13	118.8 (2)	C29—C30—H30B	108.7
C11—C12—H12	120.6	H30A—C30—H30B	107.6
C13—C12—H12	120.6	C30—C31—C32	114.43 (17)
C12—C13—C8	121.3 (2)	C30—C31—H31A	108.7
C12—C13—H13	119.4	C32—C31—H31A	108.7
C8—C13—H13	119.4	C30—C31—H31B	108.7
O3—C14—O2	123.7 (2)	C32—C31—H31B	108.7
O3—C14—C15	125.0 (3)	H31A—C31—H31B	107.6
O2—C14—C15	111.2 (2)	C33—C32—C31	114.58 (17)
C20—C15—C16	119.4 (2)	C33—C32—H32A	108.6
C20—C15—C14	118.3 (2)	C31—C32—H32A	108.6
C16—C15—C14	122.3 (2)	C33—C32—H32B	108.6
C17—C16—C15	120.4 (2)	C31—C32—H32B	108.6
C17—C16—H16	119.8	H32A—C32—H32B	107.6
C15—C16—H16	119.8	C32—C33—C34	114.73 (18)
O5—C17—C16	125.1 (2)	C32—C33—H33A	108.6
O5—C17—C18	115.3 (2)	C34—C33—H33A	108.6
C16—C17—C18	119.5 (2)	C32—C33—H33B	108.6
O4—C18—C19	124.8 (2)	C34—C33—H33B	108.6
O4—C18—C17	115.6 (2)	H33A—C33—H33B	107.6
C19—C18—C17	119.6 (2)	C33—C34—C35	115.30 (18)
C18—C19—C20	120.4 (2)	C33—C34—H34A	108.4
C18—C19—H19	119.8	C35—C34—H34A	108.4
C20—C19—H19	119.8	C33—C34—H34B	108.4
C15—C20—C19	120.7 (2)	C35—C34—H34B	108.4
C15—C20—H20	119.7	H34A—C34—H34B	107.5
C19—C20—H20	119.7	C36—C35—C34	114.29 (18)
O5—C21—H21A	109.5	C36—C35—H35A	108.7
O5—C21—H21B	109.5	C34—C35—H35A	108.7
H21A—C21—H21B	109.5	C36—C35—H35B	108.7
O5—C21—H21C	109.5	C34—C35—H35B	108.7
H21A—C21—H21C	109.5	H35A—C35—H35B	107.6
H21B—C21—H21C	109.5	C35—C36—C37	115.0 (2)
O4—C22—C23	107.48 (17)	C35—C36—H36A	108.5
O4—C22—H22A	110.2	C37—C36—H36A	108.5
C23—C22—H22A	110.2	C35—C36—H36B	108.5
O4—C22—H22B	110.2	C37—C36—H36B	108.5
C23—C22—H22B	110.2	H36A—C36—H36B	107.5
H22A—C22—H22B	108.5	C36—C37—H37A	109.5
C22—C23—C24	112.98 (17)	C36—C37—H37B	109.5
C22—C23—H23A	109.0	H37A—C37—H37B	109.5
C24—C23—H23A	109.0	C36—C37—H37C	109.5
C22—C23—H23B	109.0	H37A—C37—H37C	109.5
C24—C23—H23B	109.0	H37B—C37—H37C	109.5
H23A—C23—H23B	107.8		
			
C6—C1—C2—C3	−0.9 (5)	C14—C15—C16—C17	177.3 (2)
C1—C2—C3—C4	−0.3 (5)	C21—O5—C17—C16	−4.1 (4)
C2—C3—C4—C5	1.2 (5)	C21—O5—C17—C18	175.7 (2)
C2—C3—C4—C7	−176.2 (3)	C15—C16—C17—O5	179.0 (2)
C3—C4—C5—C6	−0.9 (5)	C15—C16—C17—C18	−0.8 (3)
C7—C4—C5—C6	176.4 (3)	C22—O4—C18—C19	1.7 (3)
C2—C1—C6—C5	1.1 (5)	C22—O4—C18—C17	−178.31 (19)
C4—C5—C6—C1	−0.2 (5)	O5—C17—C18—O4	1.2 (3)
C8—O1—C7—C4	177.7 (2)	C16—C17—C18—O4	−178.90 (19)
C3—C4—C7—O1	−146.5 (2)	O5—C17—C18—C19	−178.75 (19)
C5—C4—C7—O1	36.2 (4)	C16—C17—C18—C19	1.1 (3)
C7—O1—C8—C9	7.1 (4)	O4—C18—C19—C20	180.0 (2)
C7—O1—C8—C13	−175.0 (2)	C17—C18—C19—C20	0.0 (3)
O1—C8—C9—C10	−179.6 (2)	C16—C15—C20—C19	1.6 (3)
C13—C8—C9—C10	2.6 (4)	C14—C15—C20—C19	−176.3 (2)
C8—C9—C10—C11	−0.1 (4)	C18—C19—C20—C15	−1.4 (3)
C9—C10—C11—C12	−3.0 (4)	C18—O4—C22—C23	179.04 (17)
C9—C10—C11—O2	173.6 (2)	O4—C22—C23—C24	−176.06 (17)
C14—O2—C11—C10	122.4 (3)	C22—C23—C24—C25	179.71 (18)
C14—O2—C11—C12	−61.0 (3)	C23—C24—C25—C26	−177.71 (18)
C10—C11—C12—C13	3.5 (4)	C24—C25—C26—C27	179.49 (17)
O2—C11—C12—C13	−173.0 (2)	C25—C26—C27—C28	179.93 (18)
C11—C12—C13—C8	−1.0 (4)	C26—C27—C28—C29	−179.22 (17)
C9—C8—C13—C12	−2.0 (4)	C27—C28—C29—C30	179.90 (18)
O1—C8—C13—C12	179.9 (2)	C28—C29—C30—C31	−179.40 (18)
C11—O2—C14—O3	2.3 (4)	C29—C30—C31—C32	179.64 (17)
C11—O2—C14—C15	−174.30 (19)	C30—C31—C32—C33	179.53 (18)
O3—C14—C15—C20	−6.3 (4)	C31—C32—C33—C34	179.61 (18)
O2—C14—C15—C20	170.3 (2)	C32—C33—C34—C35	177.86 (18)
O3—C14—C15—C16	175.8 (2)	C33—C34—C35—C36	177.31 (19)
O2—C14—C15—C16	−7.6 (3)	C34—C35—C36—C37	177.5 (2)
C20—C15—C16—C17	−0.5 (3)		

### Hydrogen-bond geometry (Å, °)

**Table:**

Cg2 and Cg3 are the centroids of the C8–C13 and C14–C20 rings, respectively.

**Table:**

*D*—H···*A*	*D*—H	H···*A*	*D*···*A*	*D*—H···*A*
C6—H6···Cg2^i^	0.93	2.97	3.576 (3)	124
C22—H22B···Cg3^ii^	0.96	2.94	3.793 (2)	148

Symmetry codes: (i) *x*−2, *y*, *z*+1; (ii) *x*+1, *y*, *z*.
